# Asthma, Type 1 and Type 2 Diabetes Mellitus, and Inflammatory Bowel Disease amongst South Asian Immigrants to Canada and Their Children: A Population-Based Cohort Study

**DOI:** 10.1371/journal.pone.0123599

**Published:** 2015-04-07

**Authors:** Eric I. Benchimol, Douglas G. Manuel, Teresa To, David R. Mack, Geoffrey C. Nguyen, Jennifer L. Gommerman, Kenneth Croitoru, Nassim Mojaverian, Xuesong Wang, Pauline Quach, Astrid Guttmann

**Affiliations:** 1 Children’s Hospital of Eastern Ontario Inflammatory Bowel Disease Centre, Division of Gastroenterology, Hepatology, and Nutrition, Children’s Hospital of Eastern Ontario, Ottawa, Canada; 2 Department of Pediatrics, University of Ottawa, Ottawa, Canada; 3 School of Epidemiology, Public Health and Preventative Medicine, University of Ottawa, Ottawa, Canada; 4 Department of Family Medicine, University of Ottawa, Ottawa, Canada; 5 Institute for Clinical Evaluative Sciences, Toronto, Canada; 6 Ottawa Hospital Research Institute, Ottawa, Canada; 7 Child Health Evaluative Sciences, The Hospital for Sick Children Research Institute, Toronto, Canada; 8 Division of Pediatric Medicine, The Hospital for Sick Children, Toronto, Canada; 9 Mount Sinai Centre for Inflammatory Bowel Disease, Toronto, Canada; 10 Dalla Lana School of Public Health, University of Toronto, Toronto, Canada; 11 Department of Medicine, University of Toronto, Toronto, Canada; 12 Department of Immunology, University of Toronto, Toronto, Canada; 13 Institute of Health Policy, Management and Evaluation, University of Toronto, Toronto, Canada; 14 Department of Paediatrics, University of Toronto, Toronto, Canada; Old Dominion University, UNITED STATES

## Abstract

**BACKGROUND:**

There is a high and rising rate of immune-mediated diseases in the Western world. Immigrants from South Asia have been reported to be at higher risk upon arrival to the West. We determined the risk of immune-mediated diseases in South Asian and other immigrants to Ontario, Canada, and their Ontario-born children.

**METHODS:**

Population-based cohorts of patients with asthma, type 1 diabetes (T1DM), type 2 diabetes (T2DM), and inflammatory bowel disease (IBD) were derived from health administrative data. We determined the standardized incidence, and the adjusted risk of these diseases in immigrants from South Asia, immigrants from other regions, compared with non-immigrant residents of Ontario. The risk of these diseases in the Ontario-born children of immigrants were compared to the children of non-immigrants.

**RESULTS:**

Compared to non-immigrants, adults from South Asia had higher risk of asthma (IRR 1.56, 95%CI 1.51-1.61) and T2DM (IRR 2.59, 95%CI 2.53-2.65). Adults from South Asia had lower incidence of IBD than non-immigrants (IRR 0.32, 95%CI 0.22-0.49), as did immigrants from other regions (IRR 0.29, 95%CI 0.20-0.42). Compared to non-immigrant children, the incidence of asthma (IRR 0.66, 95%CI 0.62-0.71) and IBD (IRR 0.47, 95%CI 0.33-0.67) was low amongst immigrant children from South Asia. However, the risk in Ontario-born children of South Asian immigrants relative to the children of non-immigrants was higher for asthma (IRR 1.75, 95%CI 1.69-1.81) and less attenuated for IBD (IRR 0.90, 95%CI 0.65-1.22).

**CONCLUSION:**

Early-life environmental exposures may trigger a genetic predisposition to the development of asthma and IBD in South Asian immigrants and their Canada-born children.

## Introduction

Over the past century, the prevalence of immune-mediated chronic diseases has increased worldwide [1]. Many of these disorders are of higher prevalence in Westernized nations, with low prevalence noted in people living in South Asia [2–4]. Migrant studies have been used to disentangle the role of environmental exposures from genetic risk. Higher rates of immune disorders in migrants from South Asia to the West have suggested that environmental exposures are partially related to high prevalence observed in Westernized nations [5–7]. There is a paucity of information related to the risk of immune diseases in the children of South Asian immigrants born in Western nations.

This study was conducted in Ontario, Canada’s most populous province, which has a large foreign-born population with over one quarter of immigrants arriving from South Asia [8]. We described the incidence and prevalence of immune-mediated diseases including asthma, Type 1 (T1DM) and Type 2 diabetes mellitus (T2DM), and inflammatory bowel disease (IBD) amongst South Asian immigrants to Ontario. We also determined the incidence of these immune-mediated disorders in the Ontario-born children of immigrants from South Asia and other regions, compared to children of non-immigrants. In so doing, we aimed to determine the risk contributed by early life exposure to the Canadian environment in the development of these immune-mediated chronic diseases.

## Materials and Methods

### Study Design

We conducted a population-based, retrospective cohort study of all residents of Ontario diagnosed with asthma, T1DM, T2DM, and IBD. We compared the incidence and prevalence of these chronic diseases in immigrants from South Asia, immigrants from other regions, and non-immigrants. We also compared incidence in the children of immigrant and non-immigrant mothers. We calculated annual age- and sex-standardized incidence and prevalence and incidence per person-years of follow-up for the full period. This study was approved by the Research Ethics Board of the Children’s Hospital of Eastern Ontario, and The Ottawa Hospital. Ontario’s administrative databases are maintained by Institute for Clinical Evaluative Sciences (ICES) through a data sharing agreement with the Ontario Ministry of Health and Long Term Care. The datasets used in this study were linked using unique, encoded identifiers and analyzed at ICES, a prescribed entity under Ontario’s Personal Health Information Protection Act. This designation allows for research using encrypted identifiers without obtaining informed consent.

### Data sources

This study drew from the health administrative data of all residents of Ontario, Canada who were <65 years during the study period of fiscal years (FY—April 1 to March 31) 1994–2008 who qualified for universal government health care insurance (>99% of the population). The Ontario Asthma Surveillance Information System (OASIS) [9–10], the Ontario Diabetes Database (ODD) [11–12], and the Ontario Crohn’s and Colitis Cohort (OCCC) [13–14] are patient-derived cohorts created using validated algorithms of health care contacts to classify patients as having or not having asthma, diabetes, and/or IBD respectively. For each of the disease cohorts, validated look-back periods were used to distinguish incident from prevalent cases [9–14]. These cohorts use physician billing data (from the Ontario Health Insurance Plan database), hospitalization records (from the Canadian Institute for Health Information Discharge Abstract Database), and population data (from the Registered Persons Database (RPDB) and Canadian census information) to derive patient groups and standardized populations. Mothers and their babies are linked using a unique maternal-newborn matching number. From 2002 and earlier, the unique matching was not available, instead probabilistically linked hospital care and demographic information, yielding a linkage rate of 98.4% of all births (sensitivity 96.1%, specificity 99.2%). Immigration and visa status, age and date of arrival to Canada, country of birth, and pre-migration education level were derived from national immigration data provided by Citizenship and Immigration Canada (overall successful match rate 86.1%; of which 66.9% were matched deterministically, and 19.1% were matched probabilistically).

### Setting and participants

Patients with asthma, T1DM, T2DM, and inflammatory bowel disease were identified from health administrative data using validated classification algorithms. Full health administrative data was available in Ontario from April 1, 1991. The look-back period for asthma was five years, so incident cases could be distinguished from prevalent cases from 1996. For both diabetes and pediatric IBD, a 3 year look-back period was validated, so incident cases were available from 1994. For adult-onset IBD, an eight year look-back period was necessary, so incident cases were available from 1999. For residents born after April 1, 1991, administrative data was available for their full lifespan and therefore a look-back period was not required to distinguish incident from prevalent cases. Without sufficient data available for the look-back period, cases were considered prevalent but not incident.

We classified all residents under 65 years of age as being immigrants (those who legally immigrated to Canada after 1985) or non-immigrants (those born in Canada, or who may have immigrated before 1985 in whom immigration records were unavailable). Immigrants were defined as people who arrived to Canada with any legal status. Since the purpose of this study was to determine differences in incidence and prevalence in immigrants and their children, immigrants who arrived to Canada or were diagnosed with these disorders after their 65^th^ birthday were excluded, as they were unlikely to reproduce. We categorized the country of birth of immigrants using the World Bank classification of world regions [15]. Immigrants from South Asia were those born in Afghanistan, Bangladesh, Bhutan, India, Maldives, Nepal, Pakistan, or Sri Lanka. Immigrants from other regions were included as a comparison group. Immigrants who did not have a recorded country of birth were excluded (3.9% of all immigrants). The children of immigrant mothers were defined as those born in an Ontario hospital after 1988.

### Statistical Analysis

Incidence and prevalence were calculated in three year blocks for T1DM, T2DM, and pediatric IBD from 1994–2008, for asthma from 1996–2008, and for adult-onset IBD from 1999–2008. Point-prevalence was calculated on July 1^st^ in the middle year of the three year block. Standardized incidence and prevalence were calculated per 100,000 population, with corresponding 95% confidence intervals (CI) based on gamma distribution. We used the Canadian censuses from 1996, 2001 and 2006 to determine annual intercensal population estimates [16]. Standardized rates were calculated using age-appropriate standard populations. We also calculated overall incidence per person-years of follow-up, with 95% CI for the full periods of data availability.

To compare incidence in South Asian immigrants, other immigrants, and non-immigrants, we calculated incidence rate ratio (IRR) with 95% CI, adjusted for age and sex. We determined significant difference in standardized incidence between immigrant status groups using Poisson regression analysis. The same analyses were used for calculation of incidence and prevalence in the Ontario-born children of immigrants and non-immigrants. Overdispersion was assessed by calculating the deviance divided by the numbers of degrees of freedom. In the case of adult IBD analyses, where this value was much greater than 1, we used the negative binomial model for Poisson regression with scaled deviance.

## Results

### Characteristics of the study population

From 1985 to 2008, we included 443,265 immigrants to Ontario from South Asia, and 1,454,505 immigrants from other regions. In addition, we included 10,753,800 non-immigrants. The characteristics of the South Asian immigrants, immigrants from other regions, and non-immigrant populations are listed in Table 1. Immigrants originated predominantly from East-Asia and Pacific (25.7% of the overall immigrant cohort), South Asia (23.4%), and Latin America and the Caribbean (13.0%).

**Table 1:** Descriptive characteristics of the included population at the time of immigration to Canada, on March 31, 2009 (end of recruitment).

	At Immigration to Canada		On March 31, 2009		
	South Asian Immigrants	Other Immigrants	South Asian Immigrants	Other Immigrants	Long-term residents
**N**	443,265	1,454,505	443,265	1,454,505	10,753,800
**Median age (IQR)**	28 (17–37)	28 (15–36)	38 (29–47)	40 (29–49)	37 (18–53)
**Age under 18 (%)**	112,901 (25.5%)	416,552 (28.6%)	60,509 (13.7%)	148,166 (10.2%)	2,593,692 (24.1%)
**Male (%)**	229,112 (51.7%)	714,164 (49.1%)	229,112 (51.7%)	714,164 (49.1%)	5,422,054 (50.4%)
**Mean Neighbourhood Income Quintile**					
None specified (%)	36,931 (8.3%)	296,784 (20.4%)	16,777 (3.8%)	47,324 (3.3%)	461,611 (4.3%)
Q1—lowest (%)	183,675 (41.4%)	425,115 (29.2%)	142,899 (32.2%)	378,487 (26.0%)	1,835,136 (17.1%)
Q2 (%)	98,085 (22.1%)	265,357 (18.2%)	104,596 (23.6%)	308,665 (21.2%)	1,963,415 (18.3%)
Q3 (%)	65,182 (14.7%)	191,691 (13.2%)	89,802 (20.3%)	274,566 (18.9%)	2,048,805 (19.1%)
Q4 (%)	40,047 (9.0%)	150,329 (10.3%)	60,933 (13.8%)	256,373 (17.6%)	2,198,049 (20.4%)
Q5—highest (%)	19,345 (4.4%)	125,229 (8.6%)	28,258 (6.4%)	189,090 (13.0%)	2,246,784 (20.9%)
**World region of birth**					
East Asia and Pacific (%)		487,909 (33.5%)			
Eastern Europe and Central Asia (%)		151,519 (10.4%)			
Latin America and the Caribbean (%)		246,231 (16.9%)			
Middle East and North Africa (%)		179,674 (12.4%)			
Sub-Saharan Africa (%)		111,764 (7.7%)			
Western Europe and North America (%)		196,552 (13.5%)			
Not classified* (%)		80,856 (5.6%)			
**Immigration visa category**					
Independent (%)	175,769 (39.7%)	568,693 (39.1%)			
Family (%)	192,685 (43.5%)	640,431 (44.0%)			
Refugee (%)	52,538 (11.9%)	117,691 (8.1%)			
Other (%)	7899 (1.8%)	81,996 (5.6%)			
**Educational level at landing**					
No education (%)	62,652 (14.1%)	171,444 (11.8%)			
Secondary level or less (%)	181,637 (41.0%)	657,318 (45.2%)			
No university qualifications (%)	33,632 (7.6%)	208,804 (14.4%)			
Some university (%)	13,235 (3.0%)	65,265 (4.5%)			
University degree or higher (%)	152,102 (34.3%)	351,609 (24.2%)			
None specified (%)	7 (<0.1%)	65 (<0.1%)			
**Years of immigration**					
1985–1989 (%)	24,546 (5.5%)	219,497 (15.1%)			
1990–1994 (%)	70,605 (15.9%)	388,758 (26.7%)			
1995–1999 (%)	106,997 (24.1%)	308,466 (21.2%)			
2000–2004 (%)	163,651 (36.9%)	351,759 (24.2%)			
2005–2008 (%)	77,466 (17.5%)	186,025 (12.8%)			

*Country of birth was not included in the World Bank classification of world regions.

### Asthma

**Fig 1:**
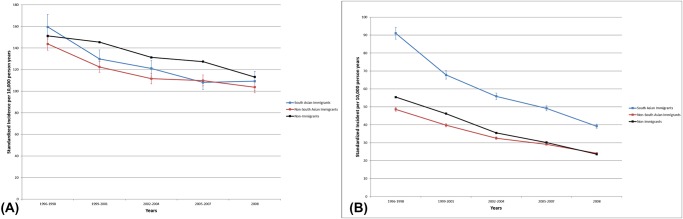
Incidence of asthma in (A) children and (B) adults 1996–2008.

Asthma incidence decreased in South Asian immigrants between 1996 to 2008, from 120.8 to 63.2 per 10,000 at-risk individuals, compared to the non-immigrant decrease from 83.0 to 51.2 per 10,000 (Fig 1). Children from South Asia had lower incidence of asthma compared to non-immigrant children (IRR 0.66, 95% CI 0.62 to 0.71) (Table 2). However, the children of women from South Asia had significantly higher incidence than the children of non-immigrants (IRR 1.75, 95% CI 1.69 to 1.81). Adults from South Asia also had higher incidence of asthma than non-immigrants (IRR 1.56, 95% CI 1.51 to 1.61).

**Table 2:** Incidence of asthma in immigrants and their children, compared to non-immigrants, for the full time period 1996–2008.

Immigration Status	Number of incident cases	Total population	Person-years	Incidence per 10,000 person-years (95% CI)	Adjusted IRR (95% CI)
Children <18y					
South Asian Immigrant	5,971	118,944	588,283	101.5 (98.9 to 104.1)***	0.66 (0.62 to 0.71)
Immigrant from Other Regions	16,702	406,078	2,086,227	80.1 (78.9 to 81.3)***	0.49 (0.47 to 0.51)
Non-Immigrant	518,579	4,674,592	31,060,703	167.0 (166.5 to 167.4)	REF
Adults 18–64y					
South Asian Immigrant	18,350	448,958	3,140,963	58.4 (57.6 to 59.3)***	1.56 (1.51 to 1.61)
Immigrant from Other Regions	43,151	1,501,647	12,260,359	35.2 (34.9 to 35.5)***	0.91 (0.89 to 0.93)
Non-Immigrant	356,553	9,163,413	87,227,249	40.9 (40.7 to 41.0)	REF
Ontario-born children					
South Asian Immigrant Mother	24,036	118,135	593,545	405.0 (399.9 to 410.1)***	1.75 (1.69 to 1.81)
Immigrant Mother from Other Regions	55,205	313,019	1,859,813	296.8 (294.4 to 299.3)***	1.27 (1.23 to 1.30)
Non-immigrant Mother	315,457	1,869,079	13,224,912	238.5 (237.7 to 239.4)	REF

NB Age groups represent age at disease onset. The category of Ontario-born children represent children born after 1991, where full administrative data is available from birth.

***P<0.0001 compared to non-immigrant group by age- and sex-adjusted Poisson regression analysis.

CI: confidence intervals; IRR: relative incidence ratio; REF: reference group

Standardized prevalence of asthma per 10,000 population increased in South Asian immigrants from 606.4 in 1996, to 652.4 in 2002, to 700.6 in 2008. Prevalence also increased per 10,000 non-immigrants, from 686.8 in 1996, to 1280.9 in 2002, to 1524.1 in 2008.

### Diabetes Mellitus

**Fig 2:**
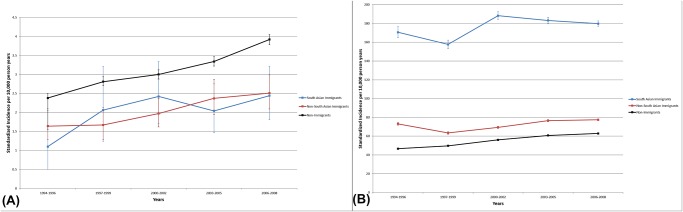
Incidence of (A) type 1 diabetes in children and (B) type 2 diabetes in adults 1994–2008.

The standardized incidence of T1DM per 10,000 South Asian immigrant children <18 increased from 0.8 in 1994, to 2.4 in 2002, to 2.7 in 2008. Incidence per 10,000 non-immigrants also increased in from 2.4 in 1994, to 3.1 in 2002, to 3.9 in 2008 (Fig 2). Standardized prevalence of T1DM per 10,000 South Asian immigrant children was 2.5 in 1994, 8.3 in 2002, and 10.9 in 2008. Prevalence per 10,000 non-immigrant children was 16.7 in 1998, 22.4 in 2002, and 28.0 in 2008. For the full 1994–2008 period, the incidence of T1DM was lower in both South Asian immigrant children and immigrant children from other regions, compared to non-immigrant children, although only the group from other regions were statistically significantly lower than non-immigrant children (Table 3). The Ontario-born children of both South Asian immigrants (IRR 0.68, 95% CI 0.46 to 0.998) and immigrants from other regions (IRR 0.68, 95% CI 0.54 to 0.85) had lower incidence of T1DM than the children of non-immigrants.

**Table 3:** Incidence of diabetes in immigrants and their children, compared to non-immigrants, for the full time period 1994–2008.

Immigration Status	Number of incident cases	Total population	Person-years	Incidence per 10,000 person-years (95% CI)	Adjusted IRR (95% CI)
Children <18y (Type 1 diabetes)					
South Asian Immigrant	167	122,300	654,197	2.6 (2.2 to 3.0)	0.84 (0.55 to 1.28)
Immigrant from Other Regions	589	429,044	2,450,813	2.4 (2.2 to 2.6)	0.79 (0.63 to 0.99)
Non-Immigrant	11,996	4,962,707	39,245,619	3.1 (3.0 to 3.1)	REF
Adults 18–64y (Type 2 diabetes)					
South Asian Immigrant	46,205	449,354	3,160,036	146.2 (144.9 to 147.6)***	2.59 (2.53 to 2.65)
Immigrant from Other Regions	76,621	1,510,808	13,041,245	58.8 (58.3 to 59.2)***	1.05 (1.03 to 1.07)
Non-Immigrant	552,153	9,355,295	98,119,708	56.3 (56.1 to 56.4)	REF
Ontario-born children (Type 1 diabetes)					
South Asian Immigrant Mother	153	118,241	737,186	2.1 (1.8 to 2.4)*	0.68 (0.46 to 0.998)
Immigrant Mother from Other Regions	467	313,543	2,261,075	2.1 (1.9 to 2.3)**	0.68 (0.54 to 0.85)
Non-immigrant Mother	4,885	1,874,134	16,036,693	3.1 (3.0 to 3.1)	REF

NB Age groups represent age at disease onset. The category of Ontario-born children represent children born after 1991, where full administrative data is available from birth.

* P<0.05,

**P<0.001,

***P<0.0001 compared to non-immigrant group by age- and sex-adjusted Poisson regression analysis.

CI: confidence intervals; IRR: relative incidence ratio; REF: reference group.

The incidence of T2DM in patients diagnosed aged 18–64 years was higher in South Asian immigrants than in non-immigrants (Fig 2). Standardized incidence per 10,000 South Asian immigrants was 198.5 in 1994, 206.7 in 2002, and 182.8in 2008. In non-immigrants, incidence was 50.7 in 1994, 60.4 in 1998, and 62.8 per 10,000 in 2008. In immigrants from South Asia, standardized prevalence per 10,000 was 704.9 in 1994, 970.7 in 2002, and 1218.0 in 2008. In non-immigrants, prevalence per 10,000 was 310.5 in 1994, 464.8 in 2002, and 581.8 in 2008. Over the 1994–2008 period, incidence of T2DM was higher in South Asian immigrants (IRR 2.59, 95% CI 2.53 to 2.65) and slightly higher in immigrants from other regions (IRR 1.05, 95% CI 1.03 to 1.07) compared to non-immigrants (Table 3).

### Inflammatory Bowel Disease

**Fig 3:**
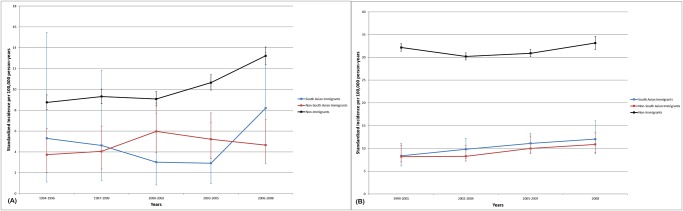
Incidence of inflammatory bowel disease in (A) children 1994–2008 and (B) adults 1999–2008.

In South Asian immigrants, standardized incidence of IBD per 100,000 at-risk individuals aged 6 months to 65 years old was 9.1 in 1999, 8.5 in 2004, and 10.4 in 2008 (Fig 3). Incidence of IBD per 100,000 non-immigrants was higher: 26.5 in 1999, 24.7 in 2004, and 28.2 in 2008. Of the main IBD sub-types, South Asian immigrants had higher incidence of ulcerative colitis (range 4.9–8.0 per 100,000) than Crohn’s disease (range 0.7–3.9 per 100,000). By contrast in non-immigrants, the incidence of ulcerative colitis (range 10.9–12.7 per 100,000) was similar to that of Crohn’s (range 11.6–12.5 per 100,000).

The incidence of pediatric-onset (1994–2008) and adult-onset (1999–2008) IBD was lower in South Asian immigrants compared to non-immigrants (Table 4). Compared to the children of non-immigrants, the incidence remained lower in the children of immigrants from other regions. However, the risk of developing IBD in the Ontario-born children of South Asian immigrants was not different from the children of non-immigrants. There was a similar risk of ulcerative colitis in the children of South Asian mothers compared to the children of non-immigrants (IRR 1.23, 95% CI 0.79–1.94). The risk of Crohn’s in the children of South Asian immigrants remained lower than the children of non-immigrants (IRR 0.39, 95% CI 0.27–0.57).

**Table 4:** Incidence of IBD in immigrants and their children, compared to non-immigrants, for the full time period available.

Immigration Status	Number of incident cases	Total population	Person-years	Incidence per 100,000 person-years (95% CI)	Adjusted IRR (95% CI)
Children <18y (1994–2008)					
South Asian Immigrant	31	112,425	627,800	4.9(3.3 to 7.0)**	0.47 (0.33 to 0.67)
Immigrant from Other Regions	103	368,777	2,159,083	4.7 (3.9 to 5.8)***	0.47 (0.38 to 0.57)
Non-Immigrant	3805	4,532,055	37,224,059	10.2 (9.9 to 10.5)	REF
Adults 18–64y (1999–2008)					
South Asian Immigrant	289	422,864	2,791,421	10.3 (9.1 to 11.5)***	0.32 (0.22 to 0.49)
Immigrant from Other Regions	853	1,306,270	9,331,817	9.1 (8.5–9.8)***	0.29 (0.20 to 0.42)
Non-Immigrant	20,635	7,987,726	65,903,239	31.3 (30.9–31.7)	REF
Ontario-born children					
South Asian Immigrant Mother	41	109,830	713,821	5.8 (4.1 to 7.8)	0.90 (0.65 to 1.22)
Immigrant Mother from Other Regions	68	271,503	2,015,218	3.4 (2.6 to 4.3)***	0.53 (0.41 to 0.67)
Non-immigrant Mother	923	1,624,120	14,396,199	6.4 (6.0–6.8)	REF

NB Age groups represent age at disease onset. The category of Ontario-born children represent children born after 1991, where full administrative data is available from birth.

* P<0.05,

**P<0.001,

***P<0.0001 compared to non-immigrant group by age- and sex-adjusted Poisson regression analysis.

CI: confidence intervals; IBD: inflammatory bowel disease; IRR: relative incidence ratio; REF: reference group.

Standardized prevalence of IBD per 100,000 South Asian immigrants increased from 78.2 in 1994, to 113.0 in 2002, to 160.3 in 2008. Prevalence also increased in non-immigrants, from 308.9 in 1994, to 476.1 in 2002, to 577.3 per 10,000 in 2008.

## Discussion

In this study, we have reported population-based estimates of incidence and prevalence of multiple immune-mediated chronic diseases in immigrants to Canada from South Asia, as well as their Canadian-born children. In so doing, we have assessed the interaction between early-life exposure to the Canadian environment and the South Asian genetic background on the risk of these disorders. Adults from South Asia had higher risk of asthma and T2DM, compared to either non-immigrants or immigrants from other regions. Adults from South Asia had lower incidence of IBD than non-immigrants, but similar rates to immigrants from other regions. Although the incidence of asthma and IBD was low amongst immigrant children from South Asia relative to non-immigrant children, rates of asthma were higher in their Ontario-born children, and the risk of IBD in Ontario-born children of South Asian immigrants was similar to the children of non-immigrants. This did not occur in the children of immigrants from other regions, who were predominantly from East Asia or the Caribbean. These findings imply a significant effect of early-life exposure to the Canadian environment on the risk of asthma and IBD, but not of T1DM for Canadian-born South-Asians.

There was a low incidence of asthma in immigrant children from South Asia, but higher rates in adult immigrants, and the children of South Asian immigrants. The higher risk of asthma in adults may be secondary to environmental or occupational exposures in South Asia, not a contributing factor in children who arrive to Canada. We have demonstrated that the incidence of asthma in the children of South Asian mothers, and those of other immigrant mothers, was significantly higher that the incidence in children of non-immigrant mothers. Early life or in utero exposures to environmental factors specific to the Canadian context may be implicated. For example, Cesarean section rates, antibiotic usage, low maternal vitamin D levels, and a hygienic environment have all been suggested to be involved [17]. All of these are more prevalent in Canada compared to South Asian and other developing nations.

Incidence of T1DM in South Asian immigrants was similar to that of non-immigrant children. Similarly, the Ontario-born children of South Asian immigrant women had similar incidence of T1DM to the children of non-immigrant women. These findings are consistent with the findings from Leicestershire, UK, which assessed incidence in British children of South Asian ethnicity, compared to Caucasians [18]. The higher incidence and prevalence of T2DM in South Asian immigrants was previously demonstrated in Ontario [19], the UK [20], Sweden [21], and other Nordic countries [22]. Genetic predisposition in the context of a Westernized diet, increased obesity, and decreased physical activity are thought to be contributory factors.

We demonstrated low risk of IBD in both children and adults from South Asia. Rates were similar in South Asian immigrants and immigrants from other regions. However, the Ontario-born children of immigrants from South Asia had similar incidence of IBD as non-immigrants. The children of immigrants from other regions retained the lower risk of IBD compared to non-immigrants. This suggests that early-life exposure to the Canadian environment results in a risk of IBD equivalent in Ontario-born South Asian and Caucasian children. This result contrasts somewhat to a study from Vancouver, Canada, which reported much higher risk of IBD in children of South Asian ethnicity [23], potentially due to ethnicity differences in the two populations. British Columbia receives a higher proportion of South Asian immigrants of Sikh origin than Ontario. Nevertheless, both studies convey the increased risk attributed to early life in Canada with the development of IBD in children. Increased rates of ulcerative colitis have also been reported in studies of adults from South Asia who emigrate to the UK [24–25] and Vancouver [26]. To our knowledge, this is the first population-based study to demonstrate low rates of IBD in South Asian immigrants to Canada, but less attenuated in their Ontario-born children, who had similar incidence as the children of non-immigrants.

Asthma, T1DM and IBD have all been associated with both the hygiene hypothesis and alterations in the gut microbiome [27–29] although there is no known common alteration in the microbiome to explain these different immune-related diseases. Considering these disorders are associated with hundreds of genetic risk alleles, the gene-environment interaction is likely complex, and may involve unknown epigenetic or triggering factors. There may be specific genetic risk modifiers for asthma and IBD which are only activated in the Canadian milieu, which may not be present for T1DM. Alternatively, the Canadian environment may play a stronger role in determining risk of asthma and IBD compared to T1DM.

The lower risk of these chronic diseases in immigrants may have been related to the healthy immigrant effect. This concept refers to the relative good health in immigrants compared to the native populations of their home countries [30]. This may be due to self-selection or external selection of relatively healthy and productive migrants by immigration authorities. However, by assessing only the risk of incidence through validated look-back periods to exclude prevalent cases, the risk of bias should have been minimized. Nevertheless, the healthy immigrant effect suggests that people who developed these diseases in their home countries could be less likely to immigrate to Canada, resulting in a low-risk population of immigrants.

The use of administrative data presents some limitations. Firstly, grouping of patients based on the World Bank economic classification resulted in regional groups with ethnically and genetically diverse populations. However, the definitions allow for adequate power to estimate risk of these rare chronic diseases in children and adults. Despite the use of identification algorithms validated in Ontario patients of appropriate age, the risk of misclassification bias is always possible when determining the epidemiology of disease using health administrative data. The accuracy of these algorithms to identify disease in immigrant and ethnic groups has not been determined, and differential accuracy may have resulted in error of relative proportions. For example, if immigrants are less likely to access health care and therefore be identified by providers as having the chronic disease in question, they may not be identified by the algorithms. This could have resulted in lower incidence of these diseases in immigrants. Finally, the diabetes billing codes do not distinguish between T1DM and T2DM, and the algorithms used an arbitrary cut-off age of 19 years to distinguish the two. However, a national surveillance study estimated that fewer than 50 cases of T2DM exist amongst Ontario children [31].

Another limitation is the availability of longitudinal data. Immigration data was only available from 1985 forward. Therefore, immigrants who arrived to Ontario before 1985 would be considered non-immigrants. Since complete health administrative data was only available after 1 April 1991, we could only include the children of immigrant women born after that date, thus limiting our analyses to a relatively young Ontario-born population. If the gene-environment interaction was strongest in adult-onset disease, we may have underestimated the role of early-life exposure on risk. Ontario health administrative data does not link records of fathers to their children, and therefore were uncertain of the fathers’ immigration status. However, the overall rate of intermarriage amongst South Asian Canadians is only 6.8%, therefore Ontario-born children of South Asian immigrant mothers likely had two South Asian parents [32].

## Conclusions

In the largest study of South Asian migrants to date, we have demonstrated a high incidence of asthma and IBD in the Ontario-born children of South Asian immigrants relative to their immigrant parents. In addition, South Asian immigrants themselves have high rates of T2DM and asthma relative to non-immigrants. While numerous environmental risk factors have been implicated in the development of these disorders, we anticipate that this study will spur the search for gene-environment interactions. Focusing our efforts on the early-life exposures of the children of South Asian immigrants may aid in the search for specific environmental risk factors for immune-mediated disorders.
